# Secondary infection with *Aeromonas hydrophila* and death of two patients with probable *Bothrops envenomation*

**DOI:** 10.1590/0037-8682-0430-2024

**Published:** 2025-07-07

**Authors:** Mariana Antunes Villa, Caroline Ogea Tavares, Gisela Cipullo Moreira, Carlos Alberto Caldeira Mendes

**Affiliations:** 1 Faculdade de Medicina de São José do Rio Preto, São José do Rio Preto, SP, Brasil.; 2 Centro de Informação e Assistência Toxicológica de São José do Rio Preto, São José do Rio Preto, SP, Brasil.

**Keywords:** Aeromonas hydrophila, Snakebite, Bothrops

## Abstract

Secondary infection following snakebites is a common complication, and few studies exist in the literature regarding the most frequent microorganisms and the most appropriate antibiotic regimens. This article reports two cases of patients who progressed to death due to infection by *Aeromonas hydrophila* following a snakebite from the genus *Bothrops* in southeastern Brazil. This bacterium is not commonly reported as an agent of this complication in the national literature; however, it should be considered for the treatment of secondary infections following snakebites because of their potentially severe progression.

## INTRODUCTION

Snakebite envenomation is a public health problem in tropical countries because of its frequency, morbidity, and mortality. In Brazil, in 2023, there were approximately 28,000 reported cases of snakebite incidents by the Notifiable Diseases Information System (SINAN)^1^, out of which - 21,000 (75%) were related to snakes of the genus *Bothrops,* belonging to the *Viperidae family.* Among this group, the main species distributed throughout the Brazilian territory are *B. atrox, B. erythromelas, B. jararaca, B. jararacussu, B. moojeni, and B. alternatus* ^2^, with the latter two being the most common species in the studied region.

Secondary infections following snakebites, such as abscesses, cellulitis, and erysipelas, are frequent complications that occur primarily after bites from snakes of the genus *Bothrops*. This is due to the proteolytic and inflammatory action of the venom, which facilitates the proliferation of bacteria following inoculation of the snake's oral microbiota^3^ ^-^ ^5^. Therefore, diagnosis and treatment involving lesion debridement, along with early and appropriate antibiotic therapy, are of utmost importance.

The present study reports two cases of victims of *Bothrops* snakebites in São José do Rio Preto, state of São Paulo. These patients died of secondary infections and septic shock caused by *Aeromonas hydrophila*. This pathogen is a facultative anaerobic gram-negative bacterium with multifactorial pathogenic potential and various virulent mechanisms. Wild animals serve as reservoirs for these bacteria because of the biofilms formed in their oral cavities^6^. Although international studies have documented a significant role of this pathogen in secondary infections following snakebites^7^, in most national studies, this pathogen has not been considered relevant^3^ ^-^ ^5^. However, in this study, both the patients died because of a secondary infection caused by *Aeromonas hydrophila*, which raises awareness about the importance of this bacterium in the context of secondary infections occurring after a snakebite. This finding presents a significant distinction compared with other reports published in the national literature.

**Ethics:** This case report was approved by the Research Ethics Committee from Hospital de Base, São José do Rio Preto, under registration number 77981424.3.0000.5415.

## CASE REPORTS

### Case 1

**FIGURE 1:**
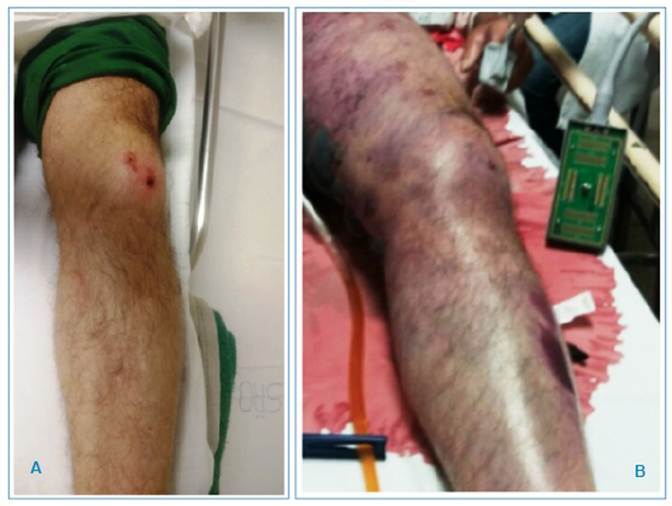
**A:** Two puncture wounds on the knee's anterior region showing ecchymosis and circumscribed edema at the bite site. **B:** Ecchymosis and blisters containing hemorrhagic fluid in the left lower limb.

A 50-year-old male rural worker suffered a snakebite on his left knee and was referred to a quaternary hospital in São José do Rio Preto, arriving 4 h after the incident. The diagnosis of snakebite caused by a *Bothrops* species was confirmed through clinical examination (two puncture wounds on the anterior region of the knee showing ecchymosis, localized pain, and initially circumscribed edema at the bite site) **(** Figure 1-A **)**. It was classified as mild, and three vials of antivenom were administered intravenously one hour after admission, along with hydration, analgesia, 12 mg of dexamethasone IV, and elevation of the affected limb. Initial laboratory results showed a complete blood count with leukocytosis (15,810/mL); a creatinine level of 1.2 mg/dL, activated partial thromboplastin time (aPTT) and prothrombin time (INR) incoagulable, a creatine kinase level of 152 UI/L, and a C-reactive protein level of 0.10 mg/dL; the urine analysis showed proteinuria and leukocyturia. 

New laboratory tests collected 11 h after antivenom administration showed significant improvement in coagulation tests (aPTT: 42.3 s; INR: 1.6), confirming antivenom therapy effectiveness; however, a worsening of renal function was noticed (creatinine: 1.7 mg/dL).

Forty-eight hours after envenomation, the patient showed worsening edema and pain in the left lower limb, with the formation of ecchymosis and blisters containing hemorrhagic fluid **(** Figure 1-B **),** as well as deterioration in renal function (creatinine: 2.2 mg/dL) and coagulation results (INR: 1.93). 

Approximately 60 h after envenomation, the patient developed hypotension, sweating, tachypnea, cyanosis, poor peripheral perfusion, and drowsiness. A hypothesis of septic shock from a cutaneous focus was made, and empirical antibiotic therapy was initiated with ceftriaxone and clindamycin. There was a worsening of laboratory results (creatinine: 3.8 mg/dL ; thrombocytopenia: 123,000/mL) and coagulation results (INR: 2.21; aPTT: 48.2 s), considered secondary to the infectious process. The patient progressed to septic shock and respiratory distress, requiring orotracheal intubation. However, despite therapeutic measures, the patient experienced cardiac arrest and subsequently died. Later, both blood cultures were positive for *Aeromonas hydrophila*, which was resistant to imipenem, meropenem, and piperacillin/tazobactam, but sensitive to amikacin, ceftazidime, ceftriaxone, cefepime, ciprofloxacin, and gentamicin.

### Case 2

A 70-year-old male patient was the victim of a snakebite to the right lower limb. He was admitted to the hospital 2 h post-incident, presenting clinical signs consistent with *Bothrops* envenomation (two punctate lesions on the right lower limb, accompanied by localized pain and edema). Envenomation was classified as mild, and three vials of antivenom were administered 1 h after admission along with hydration and analgesia. The patient had a history of Chagas cardiomyopathy and had been implanted with a pacemaker for one year. Initial laboratory examinations revealed a complete blood count showing leukocytosis (11,610/mL), a creatinine level of 1.4 mg/dL, urine analysis demonstrating leukocyturia (74,000/mL), and proteinuria. The coagulation results (aPTT and INR) were within normal limits. However, the next day, the patient exhibited worsening edema and a slight increase in INR (1.43). 

**FIGURE 2:**
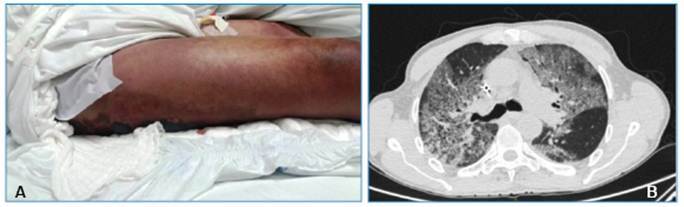
**A:** Hematoma on the right thigh. **B:** Chest CT showing ground-glass opacities in both lungs.

Four days after envenomation, the patient showed clinical deterioration, presenting with hemorrhagic blisters in the right foot and a hematoma in the right thigh **(** Figure 2-A **)**. Laboratory results indicated an elevated C-reactive protein level of 19.01 mg/dL, a white blood cell count of 6,570/mm³ with 94% segmented neutrophils, and thrombocytopenia (115,000/mm³). A diagnosis of sepsis due to a secondary infection was made, and empirical antibiotic therapy with clindamycin was initiated.

However, the patient progressed with dyspnea, desaturation, and bilateral crackles. Chest computed tomography was performed **(** Figure 2-B **)**, which led to a diagnosis of acute respiratory distress syndrome. Based on this, cefepime was added to the clindamycin regimen. The patient was intubated and transferred to the Intensive Care Unit. Approximately five days after the incident, the patient developed refractory shock and died.

Both blood cultures tested positive for *Aeromonas hydrophila*, which was sensitive to all the analyzed antimicrobials, including cefepime.

## DISCUSSION

Secondary infection following a *Bothrops* snakebite is a frequent complication, occurring in 23%⁴ to 47%^8^ of cases, and is related to the microorganisms present in the venom, oral mucosa of the snake, and skin of the individual. Incidents classified as moderate or severe, improper handling of the wound, and delays in accessing medical care increase the risk of such infection^3^ ^,^ ^9^.

Early diagnosis of abscesses is essential for patient prognosis. However, the inflammatory response caused by envenomation can impair the identification of concomitant infections because edema, pain, and local warmth can result from either acute inflammation or local infection. Nonetheless, certain clinical signs may indicate the presence of infection at the snakebite site, such as the reactivation of inflammatory symptoms in a patient with previously stable and regressing local conditions with worsening pain, sudden onset of fever or high fever, enlargement or infarction of regional lymph nodes, and fluctuations on local palpation. Treatment includes prophylactic antibiotic therapy and abscess drainage^10^.

In this study, both patients died of septic shock resulting from *Aeromonas hydrophila* secondary infection and bites from snakes of the genus *Bothrops sp*. *Aeromonas hydrophila* is a gram-negative, facultative anaerobic bacterium associated with intestinal and extraintestinal infections through the contamination of surgical wounds or animal bites^6^ ^,^ ^11^. In addition, the genus *Aeromonas* has been described in the literature as being associated with a variety of skin and soft tissue disorders, most commonly affecting healthy individuals who sustained injuries in aquatic environments upon exposure to contaminated water^12^. 

Regarding antimicrobial susceptibility, *Aeromonas sp.* have been described in the literature as resistant to ampicillin, penicillin, and first-generation cephalosporins, with increasing resistance to beta-lactams and greater susceptibility to third- and fourth-generation cephalosporins, aminoglycosides, and fluoroquinolones^6^. This aligns with the findings of the present study, as in the first case described, *Aeromonas hydrophila* was resistant to imipenem, meropenem, and piperacillin/tazobactam, but sensitive to amikacin, ceftazidime, ceftriaxone, cefepime, ciprofloxacin, and gentamicin; whereas in the second case, the bacterium was sensitive to all analyzed antimicrobials.

In Brazil, the Ministry of Health recommends the use of chloramphenicol or clindamycin combined with an aminoglycoside in cases of secondary infection following snakebite^2^. The indiscriminate use of antibiotics promotes bacterial resistance; therefore, in confirmed infections, antibiotic therapy should ideally be based on the susceptibility profile of the identified microbial agents through culture, which is not always feasible. Consequently, the choice of empirical antibiotics should be based on the predominant oral flora of snakes in this region^9^.

The literature presents a variety of results regarding the microorganisms most detected in secondary infections following snakebites. A recently published systematic review noted that the most frequently reported microorganisms in these infections were: *M. morganii, Proteus sp., S. aureus, Enterococcus sp., A. hydrophila,* and *E. coli* ^9^. The microbiota involved in secondary infections following snakebites differ from the microorganisms typically associated with erysipelas and cellulitis, which are usually caused by group A beta-hemolytic *Streptococcus* or methicillin-sensitive *Staphylococcus aureus*. Accordingly, the treatment of erysipelas and cellulitis includes first- or second-generation cephalosporins in mild cases, whereas moderate-to-severe cases are managed with intravenous crystalline penicillin and oxacillin^13^.

Regarding *Bothrops* envenomation, only one recent study from southeastern Brazil identified *A. hydrophila* as the main bacterium associated with secondary infections (four of seven analyzed)^8^. However, most national studies have not detected *A. hydrophila* as a common pathogen, neither in studies of venom nor in the oral flora of snakes, nor as a component of cultures collected from patients^3^ ^-^ ^5^. In contrast, a study conducted in Martinique on *Bothrops* lanceolatus found this bacterium to be the most prevalent^7^.

Many studies do not recommend the use of amoxicillin with clavulanate for secondary infections following snakebite incidents, despite its recommendation for infections caused by bites from other animals^4^ ^,^ ^5^ ^,^ ^9^. For example, a study conducted in Martinique^7^ concluded that antibiotic therapy with third-generation cephalosporins yielded better results than amoxicillin with clavulanate for snakebites involving *Bothrops lanceolatus.*

A study in the Brazilian Amazon showed that clindamycin was the most used first-line antibiotic; however, approximately 25% of the patients did not respond to this treatment. This highlights the importance of implementing specific guidelines to standardize the clinical management of these patients⁴. In another study conducted in the Brazilian Amazon, the use of amoxicillin with clavulanate was ineffective in preventing secondary infections caused by bites of this snake species⁵.

Thus, antibiotic therapy for secondary infections following snakebites should include gram-negative, gram-positive, and anaerobic bacteria. Recent studies have indicated that the use of amoxicillin with clavulanate is ineffective due to bacterial resistance; however, there is no consensus in the literature regarding the best empirical antibiotic regimen. Nevertheless, the literature has concluded that it is important to use broad-spectrum antibiotics for these infections. Some authors recommend a single option such as third-generation cephalosporins, metronidazole, clindamycin, or fluoroquinolones, whereas others advocate a combination of these antibiotics^6^ ^,^ ^9^. The literature also reports the empirical use of oral ciprofloxacin or levofloxacin in the treatment of *Aeromonas* infections, highlighting the advantage of fluoroquinolones, owing to the possibility of transitioning from intravenous to oral therapy^12^. It is important to emphasize that whenever feasible, the infectious agent should be isolated and its antibiotic susceptibility profile identified before starting antibiotic therapy. 

In conclusion, *Aeromonas hydrophila* is an important pathogen in cases of secondary infection resulting from snakebites due to its incidence. Therefore, the prescription of antimicrobials with coverage for this bacterium should be considered, given its antimicrobial resistance and the potentially severe progression of these infections. For empirical therapy, this also implies the need to consider not only gram-positive pathogens from the human skin but also gram-negative bacteria and anaerobes from the mouth of the snake, which may also vary according to the snake species^14^.
